# Learning from Nature: Bacterial Spores as a Target for Current Technologies in Medicine (Review)

**DOI:** 10.17691/stm2020.12.3.13

**Published:** 2020-06-28

**Authors:** B.G. Andryukov, A.A. Karpenko, I.N. Lyapun

**Affiliations:** Leading Researcher, Laboratory of Molecular Microbiology; G.P. Somov Institute of Epidemiology and Microbiology, 1 Selskaya St., Vladivostok, 690087, Russia; Professor, Department of Fundamental Sciences; Far Eastern Federal University, 10 Village Ayaks, Island Russkiy, Vladivostok, 690922, Russia;; Senior Researcher, Laboratory of Cell Biophysics; A.V. Zhirmunsky National Scientific Center of Marine Biology, Far Eastern Branch of the Russian Academy of Sciences, 17 Palchevskogo St., Vladivostok, 690041, Russia; Researcher, Laboratory of Molecular Microbiology G.P. Somov Institute of Epidemiology and Microbiology, 1 Selskaya St., Vladivostok, 690087, Russia

**Keywords:** bacterial spores, biotechnologies, sporulation, exospores, endospores, atomic-force microscopy, Raman spectroscopy, plasma chemical techniques.

## Abstract

The capability of some representatives of *Clostridium spp*. and *Bacillus spp*. genera to form spores in extreme external conditions long ago became a subject of medico-biological investigations. Bacterial spores represent dormant cellular forms of gram-positive bacteria possessing a high potential of stability and the capability to endure extreme conditions of their habitat. Owing to these properties, bacterial spores are recognized as the most stable systems on the planet, and spore-forming microorganisms became widely spread in various ecosystems.

Spore-forming bacteria have been attracted increased interest for years due to their epidemiological danger. Bacterial spores may be in the quiescent state for dozens or hundreds of years but after they appear in the favorable conditions of a human or animal organism, they turn into vegetative forms causing an infectious process. The greatest threat among the pathogenic spore-forming bacteria is posed by the causative agents of anthrax (*B. anthracis*), food toxicoinfection (*B. cereus*), pseudomembranous colitis (*C. difficile*), botulism (*C. botulinum*), gas gangrene (*C. perfringens*).

For the effective prevention of severe infectious diseases first of all it is necessary to study the molecular structure of bacterial spores and the biochemical mechanisms of sporulation and to develop innovative methods of detection and disinfection of dormant cells. There is another side of the problem: the necessity to investigate exo- and endospores from the standpoint of obtaining similar artificially synthesized models in order to use them in the latest medical technologies for the development of thermostable vaccines, delivery of biologically active substances to the tissues and intracellular structures. In recent years, bacterial spores have become an interesting object for the exploration from the point of view of a new paradigm of unicellular microbiology in order to study microbial heterogeneity by means of the modern analytical tools.

## Introduction

Bacteria are divided into gram-positive and gram-negative depending on the presence of the outer membrane and thickness of the peptidoglycan layer. Under unfavorable environmental conditions, some gram-positive bacteria realize their capability of forming spores. These spherical or slightly elongated dense structures are extremely resistant to the physical and chemical factors of the environment and are recognized as the most stable form of life on the planet [1, 2]. Since spores are formed inside the mother cell (sporangia), they are called endospores. The capability of the pathogenic microorganisms to form endospores accounts for their wide spread. In some cases, the spores of a disease causative agent are able to be independent infectious pathogens [1, 3, 4].

For example, spores of the anthrax causative agent *Bacillus anthracis* can survive in soil for decades preserving potential epizootic and epidemiological danger but having appeared on the surface they may become a source of contamination for humans and animals. For this reason, anthrax burial sites maintain the status of restricted access areas for a long time [2, 4]. Of special concern is the possibility of *B. anthracis* spores to be one of the most probable agents of bioterrorism [4–6]. A tragic incident of intentional dissemination of anthrax spores happened in the USA in 2001 (spores were mailed in the powder form in envelopes) demonstrated an evident easiness for the spores of these dangerous pathogen to become the cause of infectious disease outbreak [3, 5, 7].

Investigations of the outer architectonics and morphological spore structure in various spore-forming bacteria have revealed some specific features [2, 4, 6]. According to the structure, endospores are divided into two categories: those enveloped in a dense outer coat, exosporium, and those without it. For instance, soil bacteria *Bacillus subtilis* are the most studied example of spore formation producing no exosporium [3, 5, 7]. Though enough knowledge about the structure and function of endospores have been accumulated by microbiologists during decades, quite a number of questions in relation to exospores remain unanswered until now [5, 6—8].

The majority of data about molecular mechanisms of formation of spores and their functions in spore-forming gram-positive bacteria were studied and well described on the strains of *Bacillus spp*. and *Clostridium spp*. using mainly molecular genetic methods and transmission electron microscopy [3, 5, 8]. For example, for many years isolates of *B. subtilis* have been and remain one of the most common models for the study of genetic regulation and biochemical structural organization of the bacterial spores [3, 6, 9]. Additionally to the already mentioned *B. anthracis*, special attention among pathogenic spore-forming bacteria is attracted by *Bacillus cereus* causing toxicoinfections and the representatives of the *Clostridium spp.* genus: *C. botulinum* (causative agent of botulism), *C. perfringens* (etiological agent of gas gangrene), and *C. difficile* (the most frequent source of nosocomial infections in the world) [8–11].

The urgent character of studying the structure and spatial organization of these dormant bacterial forms by means of the current analytical technologies of molecular biology is connected primarily with their epidemiological significance.

Highly sensitive and rapid diagnostic technologies for bacterial spore detection are presently being searched for which is a burning problem for medical diagnosis, epidemiology, and for the solution of biosafety tasks as well. Besides, unsolved are the questions of effective sterilization of medical equipment and healthcare products which is important for prevention of nosocomial infections caused by pathogenic spore-forming agents [3, 6, 7].

The other perspective innovative biotechnological direction is an attempt to copy artificially the architectonics of bacterial spores to impart similar stability to the model analog systems which may find a wide scientific and practical application in pharmacology, medicine, ecology, agriculture, and other branches [8, 12].

In the present review, the results of studying molecular mechanisms of bacterial spore stability using modern analytical tools have been assessed, the directions of the application of acquired knowledge in the current technologies have been also defined.

The sources were searched using the Cochrane Library resources (Wiley Online Library directory), EMBASE (EMBASE.com), PubMed, PubMed Central, EMBASE and MEDLINE integrated on the platform Elsevier, CINAHL, Web of Science Core Collection (Science Citation Index Expanded). The sampling strategy was determined by the retrieval of the scientific papers contained in the subject directories. The depth of search covered 2003–2019 years.

## Endospores and molecular mechanisms of their stability

### Sporulation in gram-positive bacteria.

Early classifications of bacteria were based on their capability to sporulation [13]. Spore formation in bacteria was developed as a defense mechanism increasing considerably the capacity to survive in the environment. Bacterial spores can withstand unfavorable external factors such as high or low temperatures, absence of a nutrient substrate, the effect of antibacterial drugs and disinfectants, radiation, high pressure, ultraviolet radiation [14–17].

A stepwise process of spore formation is rather complicated, represents a successive genetic regulation of the synthesis of numerous specific proteins accompanied by cellular differentiation and taking about 8 h [15, 18, 19]. The outcome of sporulation is formation of mature, metabolically quiescent and reproductively inactive endospore which morphologically and structurally differs radically from the mother cell [20–22].

### Molecular genetic regulation of spore formation.

Sporulation is a genetically determined process of forming a spore inside a vegetative mother cell which is in the stationary phase of its life cycle. Exposure to stress factors triggers the genetic mechanisms providing the synthesis of transcription factors. They are responsible for bioregulation and succession of morphofunctional changes which ultimately result in the transformation of the vegetative form into a spore [11, 23—26].

**Figure 1:**
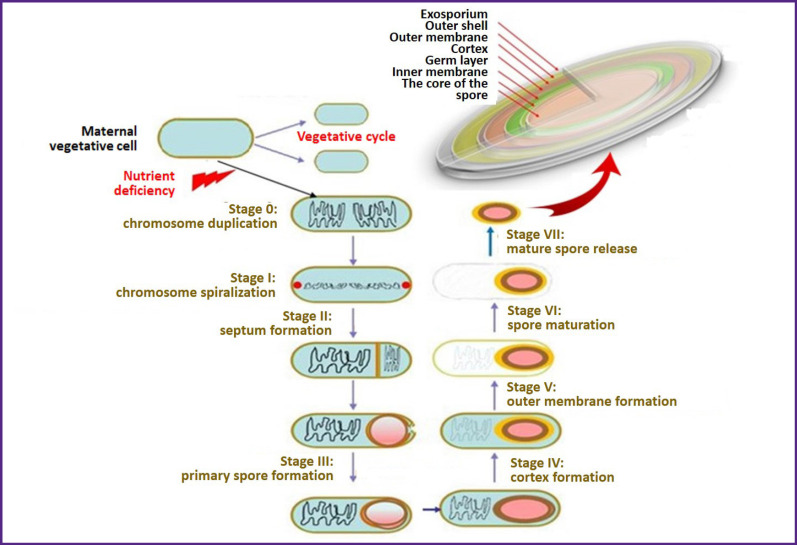
Succession of morphologic changes at different stages of sporulation in gram-positive bacteria (drawing courtesy of the authors)

Regulation of spore formation and the sophisticated molecular genetic systems providing this process were disclosed, to a large extent, only at the beginning of the XXI century [13, 27—29]. Absence of the nutrient substrate is a triggering factor of sporulation. Under these conditions, activation of gene *spo0A* encoding the similarly-named protein molecule takes place [19, 30, 31]. As a result of phosphorylation reaction, the protein Spo0A is transformed into the active form Spo0A~Р and causes expression of numerous genes (about 200) including *sigH*, *spoIIE*, *ftsZ*, and others. All these genes encode protein synthesis providing a successive running of seven sporulation stages (Figure 1). Gene *sigH*, in particular, modulates synthesis of the protein transcription σ-subunits (σН, σF) regulating the correct staging of spore formation [31–33]. This regulation pattern gives definite signals of gene expression in the mother cell and the frontal part of the spore which controls the sporulation (it requires several hours to complete) and turns on activation of a set of alternative σ-factors. These factors impart unidirectionality to the cascade culminating in endospore formation [29, 34].

As a result, the cell undergoes a very complicated, precisely definite succession of morphological and biochemical events which eventually lead to the formation of mature endospores. A special protein forces one full copy of DNA into a future prospore utilizing the ATP energy, and the division is terminated. Once the spore formation is completed, the mother cell is subjected to the programmed autolysis releasing the mature spore to the environment [35].

### Molecular mechanisms of spore thermal stability.

Thermal stability is of key significance for endospore preservation distinguishing them from vegetative cells. The mechanisms of endospore heat resistance are very complicated and are not yet studied to the full extent [15, 22, 36, 37].

As a rule, thermal sterilization is a method of choice to provide reliable sterilization. However, investigations conducted by Scheldeman et al. [38] and Schubert et al. [39] have shown that endospores are capable to survive after the standard thermal sterilization methods and special modes of thermal treatment are necessary for their elimination.

The basis of tolerance to high temperatures is known to be dehydration of the dormant cell [15, 37]. The result of sporulation is formation of endospores being in the state of reproductive and metabolic quiescence, reduced enzymatic activity, and content of high energy compounds (ATP and NADH). Several additional coatings in contrast to a common vegetative cell serve as a barrier against penetration of water substances dissolved in it. Dehydration preserves the proteins within the spore from denaturation at extremely high temperatures and irreversible aggregation which mediates the capacity of the spores to survive for a long time (decades, hundreds, and more years) without any nutrient resources under the conditions fatal for the vegetative cells [22, 36, 37, 40].

Two variants of equipment and instrument sterilization are conventionally employed in medical facilities. The first method uses steam. The objects are treated with steam heated to 110°C for 20 min. In the second variant, items are kept in a dry-air sterilizer at 120°C or higher not less than 45 min. Nevertheless, the mentioned works [38, 39] demonstrate insufficient effectivity of the thermal processing methods as some endospores are able to survive. For instance, it takes 3 h to kill spores with dry heat at 110°C and 1 h at 140°C. The researchers believe that more effective methods of sterilization are necessary to eliminate the dormant bacterial forms completely.

**Figure 2:**
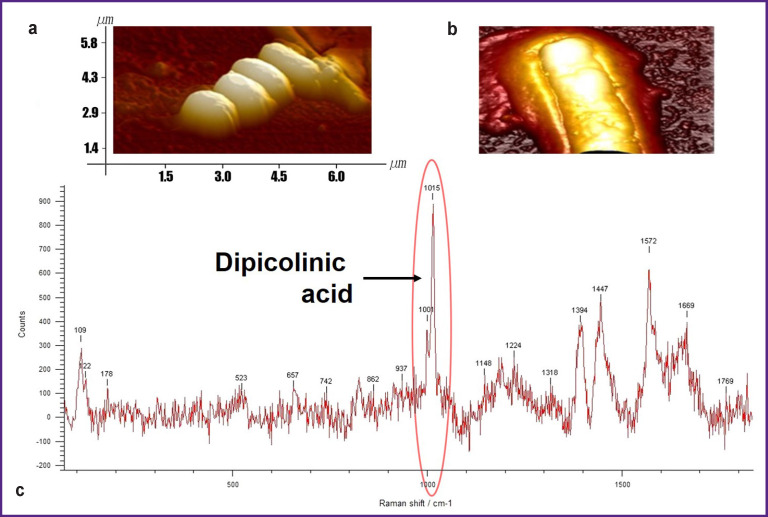
Visualization and Raman spectroscopy of the spores: (а), (b) *B. subtilis* endospores (atomic-force microscopy); (c) Raman spectrum of *B. subtili* endospore and “dipicolinic acid” biomarker (photo and drawing courtesy of the authors)

Endospores are dehydrated with dipicolinic (pyridine-2,6-dicarboxylic) acid (DPA) which forms a chelate complex with calcium and other bivalent cations and decreases water content in the spores to a very low level [7, 15, 22, 37, 41, 42]. DPA makes up 5–15% of the total endospore mass and is not found in the vegetative cells [15, 40, 43, 44] (Figure 2).

The role of DPA in a bacterial cell has not been completely understood until now though this substance was detected long ago and since that time has been actively studied [22, 41, 42, 44]. In addition to the key role in providing the cell dehydration [36], DPA participates actively in maintaining the metabolically inactive state [7] and stabilization of intracellular proteins [7, 36, 37, 41]. In this case, the cell appears to be reliably protected against extreme thermal effect since the processes of denaturation, aggregation, and lysis occur in the period prior to vegetative growth [43, 44].

Driks [45] and Setlow [29] believe that inclusion of DPA in the structure of the molecular DNA and RNA promotes formation of a gel-like polymer matrix and the stability of nucleic acids increases under the conditions of hyperthermia.

When external conditions become favorable, spore germination and generation of the vegetative bacterial forms are initiated. The DPA in this case is released to the external environment while water enters the cell triggering metabolic reactions [22, 36].

The unique properties of DPA endospores allowed modern biotechnologists to consider it as a perspective filling (excipient) for enhancing the stability of liquid biopharmaceutical highly concentrated protein preparations (e.g., antibodies) to improve their efficacy and to prevent nonspecific aggregation [37]. Besides, in recent years, DPA is considered to be a unique and reliable marker of bacterial endospores and therefore is of great interest to the designers of colorimetric and fluorometric diagnostic systems [22, 41]. Presently, the method of combination scattering (coherent Raman spectroscopy) is becoming more and more popular for reliable, prompt, and unambiguous on-line identification of this molecular marker [22, 43, 46–49].

### Molecular structure and morphology of endospores.

Formation of the structurally complicated cellular wall goes on still at the stage of a primordium (prospore) inside the mother cell. The inner coat of the daughter cell is peptidoglycan which later, after the spore germination, becomes the basic cellular wall. A cortex is built over it being an additional layer of peptidoglycan which is a bit different in its composition from the inner layer [13, 23, 25, 50]. In particular, there are modified lateral peptide and muramiko-δ-lactam crosslinks between glycan chains [15, 41, 51, 52].

Tight protein coats are formed outside and the number of them varies in different bacterial species. The study of the chemical structure of the spore outer coat, exosporium, has been difficult for a long time due to a tight cross-linking of the protein molecules. It is owing to these strong links that a dormant form acquires stability to hydrolases and other aggressive external factors. After the introduction of mass spectroscopy, presence of 70 various proteins has been established in the outer coat [51, 53, 54].

All mentioned structures are characterized by low permeability for water and other inorganic and organic molecules. Due to the specific assembly and special arrangement of the coat molecules, endospores can maintain viability for a long time waiting for the favorable environmental conditions to germinate [7, 15, 55, 56].

The spore protoplast is represented by dehydrated cytoplasm harboring the chromosome material and protein synthesizing systems. The chemical protoplasm structure contains nucleic acids, dipicolinates, ions of magnesium, calcium, manganese, and SAPSs (small acid-soluble proteins). Their molecular mass is below 12 kDa, SAPSs making up 20% of all spore proteins [20, 22, 34, 36]. Acid-resistant proteins perform primarily a protective function preventing a destructive effect of ultraviolet radiation and free radicals on the spore [20, 53, 54]. At the stage of germination, SPASs are used as a donor of amino acids [7, 54].

### The effect of environmental factors on sporulation.

Acting as a protective device for the survival of bacterial population, spores mediate the mechanisms providing their own extreme stability and maintaining the integrity of macromolecules (proteins, DNA) in the period of long quiescence. The structural endospore details (e.g. the arrangement of outer coatings) and their chemical composition may differ in various bacteria due to the diversity of the molecular mechanisms of their formation and the effect of the environmental factors on the effectiveness of sporulation [48, 50].

For instance, the effectivity of sporulation in the isolates of *B. subtilis*, *B. anthracis*, and *B. cereus* was maximal at optimal values of oxygen concentration, ionic medium composition, temperature, and pH while changes in these parameters resulted in the longer process of spore formation till its complete inhibition and to the reduction of their thermal resistance as well as the resistance to chemical and physical effects [5, 48, 50, 57]. Bressuire-Isoard et al. [57] showed that *B. cereus* spores formed at nonoptimal temperatures had a lower degree of dehydration and were less tolerant to wet heat. There are also other factors influencing the effectivity of sporulation. For example, Widderich et al. [31] found that high salinity (about 7%) inhibits sporulation of *B. subtilis* at the early stage due to the impairment of coordination of σ-factor switching and blockage of signals of gene expression in the mother cell regulating the process of spore formation.

### Molecular mechanisms regulating germination of bacterial spores.

Of great scientific and practical interest is not only spore formation but the reverse process of spore germination which is also very sophisticated from the point of view of the molecular mechanisms regulating all successive stages. The process of germination is triggered in the external conditions favorable for a specific bacterial species. The most favorable medium for pathogenic microorganisms is an animal or human organism where they may develop, proliferate, and cause infectious diseases. *B. anthracis* endospores, in particular, transfer to the active form when they get into the lung tissue and cause a pulmonary form of anthrax [24, 58—60].

The mechanism of bacterial spore germination is initiated when nutrient substrates appear in the environment: carbohydrates, amino acids, purine bases, and when there is suitable humidity, aeration, and temperature. This process runs in several stages which are strictly regulated by the biochemical mechanisms [36, 42, 61]. Substances constituting the basis of bacterial nutrition irritate the receptor apparatus of the outer endospore membrane. The receptors, in their turn, activate the process of germination which becomes irreversible at a definite stage. The initial stage of germination is marked by the release of calcium dipicolinate, ions of Н^+^, Mn^2+^, Ca^2+^, Mg^2+^. At this moment water is getting inside the cell, the pH value rises to 7.7, the glycopeptide complex is hydrolyzed [36, 60, 61].

Concurrently with the changes in pH, intracellular enzymes are activated, the metabolic processes and protein synthesizing mechanism are triggered. The cell is actively growing, swelling, increasing in volume [5, 42, 57, 62]. Once the intracellular pressure reaches the critical level, the endospore coating ruptures and a vegetative bacterial form emerges out of the spore coat [56].

These genetically determined molecular mechanisms of spore germination lie at the basis of the fractionated sterilization method (tyndallization): an object is exposed to steam at 100°C for half an hour. It is left then for a day at a temperature optimal for the growth and multiplication of microorganisms. Then the object is heated again with hot steam. This procedure is repeated several times. Bacteria managed to get out of the dormant state are destroyed affected by hyperthermia [42, 55].

In some bacteria, the process of germination has some supplementary specific features. It concerns the microorganisms capable of forming exosporium, the outermost spore coat, having specific morphofunctional characteristics.

## Morphofunctional characteristics of exosporium

### Spacial exosporium structure.

As mentioned above, in the process of their sporulation some endospores of gram-positive bacteria form an additional outer protein layer, exosporium (exospore), which serves as a barrier between the exosporium and environment [21, 63, 64].

Neither Robert Koch who described first “a globular vitreous mass” (as cited in [21]) surrounding a spore, nor Karl Flügge who suggested the name “exosporium” (as cited in [14]) did not think that this “light narrow ring surrounding spores” (R. Koch) would appear to be such a complicated and interesting object with multi-vector functions when studied with the help of state-of-the-art methods of molecular biology.

In recent years, exosporium was investigated mainly on the isolates of three basic closely related species of spore-forming gram-positive bacteria (type *Firmicutes*) belonging to the group *B. cereus* sensu lato: *B. anthracis*, *B. cereus*, and *B. thuringiensis* (bacterial insecticide) [3, 64—67]. Besides some species of *Clostridium spp*. also form exospores [9, 32, 68–73].

The exospore architecture of bacteria of the mentioned groups does not significantly differ and has similar morphology [32, 69, 74]. Usually, it represents a flexible but tight coating, a thin continuous protein basal layer, which has external hairy and internal crystalline layers. Thin structures of the exosporium may change under various growth conditions and in different bacterial species [14, 66—68].

The application of the modern methods of molecular biology allowed scientists to expand the notion of the biochemical structure and spatial organization of exosporium. In bacteria of *Bacillus spp*. and *Clostridium spp.* family, exosporium has a thin and flexible coating structure whose volume is usually larger than that of the endospore being inside [5, 57, 67].

As have been already indicated, the main structural element of exospores in various bacterial species is a thin crystalline basal protein layer [65, 70, 75–77]. The outer surface of the basal layer is coated by a hairy nap represented by collagen-like filaments, protein BclA, in the exosporium of bacteria of the *B. cereus* sensu lato group [32, 78—82]. This glycoprotein turned out to play a significant role in the protection of spores against phagocytosis [78, 83—85]. Besides, it has been proved to mediate the mechanism of immune inhibition which promotes the preservation of spores in the mouse lungs [80, 86, 87].

In bacteria of *B. cereus* sensu lato group, the main elements of exosporium structure appeared to be similar [78, 81, 84]. As a rule, exospores have irregular balloon-like shape with multiple deformations, folds, and an endospore located inside.

The area between the basal layer of exosporium and the outer endospore coat is called an interspace [70, 80] and is equal to 500 nm in *B. anthracis* [77, 81]. In some places, the basal layer is located in the immediate proximity to the outer layer of the endospore coat. The basal layer of exosporium is about 12–16 nm thick (*B. anthracis*) and is likely to consist of two sublayers about 5 nm thick [20, 67, 85].

Besides, the basal layer has a crystalline structural organization with a 6-fold symmetry and a 7 nm periodic interval. The outer surface of this structure consists of a series of hexagonal concave cups arranged in a honeycomb pattern with open ends oriented outwards [67, 84, 85]. Semipermeable channels of 20–34 nm in diameter are located between the cups [20, 67] which is sufficient for penetration of low-molecular substances but too small for the diffusion of large molecular weight proteins. Such channel arrangement imparts the barrier properties to exosporium [20, 67, 84].

The exospore nap filaments composed usually of BclA protein are 14–70 nm long and cover the entire surface of the outer coat of the basal layer [32, 84, 86–88]. The investigations [84, 88—91] showed that unlike other bacteria of *B. cereus* sensu lato group, the nap filaments in the exosporium of *B. megaterium* (strain QM B1551) were localized only on one pole and consisted of the BclA and BclB orthologous proteins and BxpB (ExsFA) protein.

### Synthesis of exosporium and its biochemical structure.

The biochemical structure of exosporium differing substantially from that of endospores was the subject of numerous investigations [14, 24, 88, 92]. A general process of its biosynthesis has been illustrated on the conceptual “bottle cap” model and in respect to *B. anthracis* includes two stages [89–91]. Synthesis initiation occurs on the central pole of the spore. At first, a germ of the future exospore appears in the spore-forming mother cell as a small laminated structure. The exospore is assembled by the subsequent deposition of proteins: it begins with the synthesis of the “cap”, the area located near the central pole of the mother cell including about 25% of the basal layer surface, and terminates by the formation of the remaining “bottle” part of the exosporium (about 75%). Synthesis is completed on the opposite spore pole by attaching the exospore basal layer to the outer coat of the spore. The studies conducted in recent years on the strains *B. anthracis* and *B. cereus* have shown that a number of proteins are involved at different stages of separate part biosynthesis, which ultimately mediates its sophisticated biochemical structure [60, 75, 92–94].

Investigating the mutant isolates of *B. anthracis*, Boydston et al. [74] and Steichen et al. [16] were the first to find that the basal layer of the exosporium does not represent a biochemically uniform structure: its “cap” and “noncap” areas differ in the protein composition. Thus, CotY is the protein specific for the “cap” while proteins BclB, ExsY, ВхрВ (also known as ExsFA), ExsFВ, and BclA are specific for the rest area of the exosporium [16, 74, 75, 92]. Of these proteins, CotY and ExsY participate in the biosynthesis of exosporium at the initial stages of forming the basal layer basis, whereas ExsFA and ExsFВ at the final stages [74, 88]. ExsFA (exosporium protein with a mass of 17 kDa) is detected both in and beyond the “cap” area while its analog, BxpB, is mainly revealed in the “noncap” part of the exosporium [82, 90, 92]. Both these proteins (some authors put a sign of equality between them) are necessary at the stage of forming filaments of the nap layer [16, 67, 82, 90].

Among the proteins involved in the exosporium biosynthesis, a collagenic glycoprotein BclA deserves special attention. Experimental studies performed by Steichen et al. [16] and Brahmbhatt et al. [76] on the model of the recombinant glycated protein BclA of the *B. anthracis* isolate have found that this glycoprotein provides general hydrophobicity of exospores and also possesses immunodominant properties [16, 76, 77, 92]. This determines the potential capability of using BclA for the development of a vaccine against anthrax. In the experiments conducted, a specific antigen rBclA increased the defense of mice infected with *B. anthracis* spores and enhanced their phagocytosis decreasing concurrently the capacity of spore germination within macrophages which is known to be the key mechanism of anthrax pathogenesis [77, 78, 88, 92].

In the interface between the exo- and endospore, protein molecules CotY, ExsA, ExsB, ExsM, ExsY are detected which interact with the CotE endospore protein. These proteins ensure strong exospore anchoring [33, 57, 66, 74, 88] and determine its chemical structure [66, 93—95].

During the exosporium coat synthesis, protein molecules are not involved in the process simultaneously. BclA and ВхрВ proteins are formed 4–5 h after the onset of spore formation. They are synthesized in the mother cell as a complicated high molecular weight structure. Then the proteins are divided into monomers and 60 min later the process of exosporium synthesis is triggered [89]. An interesting fact is that BclA and ВхрВ proteins are linked with each other due to the formation of the covalent bonds and thereafter the process of glycosylation is initiated [89, 90]. The bonds generated between these proteins are so strong that withstand boiling in the presence of 8 M urea, 1% SDS, and reducing agents [89, 91].

Investigations carried out by Manetsberger et al. [18, 54] demonstrated that the cotW and cotX proteins encoded by the similarly-named genes are the main components of the basal layer of *B. megaterium* exospore just like the proteins CotY and ExsY in the structure of *B. cereus* and *B. anthracis* [18, 54, 74, 88, 95]. In the spores of *B. subtilis* bacteria species, these protein molecules compose a tight outer coat which is likely to be an exospore rudiment [62, 96].

There are also works disclosing the most delicate molecular structure of exospores. Thompson et al. [90, 91] showed in their research the separation of the basal part of the coat into two (or possibly more) sublayers: an internal one with glycoproteins CotY and its paralog ExsY and an external sublayer including proteins ВхрВ and ExsFВ.

The location of the definite protein molecules in different parts of the cell influences directly its properties [42]. For example, the BclA protein localized in the outermost structures protects the cell against phagocytes [84, 87] and immune agents [86, 97]. Exosporial enzymes inosine-hydrolase and alanine racemase [85, 88, 94] inhibit untimely spore germination [98, 99].

One of the candidates for a key protein component determining the structure and physiology of exosporium is ExsY. This is confirmed by the researches of Jiang et al. [26] and Terry et al. [63]. The investigators came to the conclusion that exosporium has hexagonal, honeycomb structure due to the ExsY protein. The authors believe that it becomes possible owing to the formation of the disulfide bonds between the cysteine of the protein chain and its subunits [21, 26, 63]. These bonds impart flexibility to the coat of *Bacillus spp*. and *Clostridium spp*. and at the same time high resistance to the external factors. Besides they make it possible to form the folds to provide a strong sticking to the surrounding objects [74, 88]. The ability to adhere is known to be one of the key factors of virulence in this bacteria species [8, 9, 20].

But it is not so unambiguous in relation to the bacteria of *Clostridium* genus. Interesting studies of the *C. difficile* exospore structure, the leading agent of nosocomial infection, were carried out by Calderón-Romero et al. [10]. They observed two morphotypes of exosporium differing in thickness in the endospores of these *Clostridium* genus representatives [9, 10, 69, 70, 74]. The external hairs were present in the strains of *C. difficile* in any case [9, 10, 69]. Analyzing the reasons of this phenomenon the authors suggested that the strains with different exospore thickness perform various functions during the development of the infection disease [10, 69, 70].

The same researchers found 184 proteins in the exosporium layer using a gel-free approach for its analysis and combined methods of extraction [10, 70]. Some of the identified proteins turned out to be immunogenic (BclA, CdeC, CdeM, CotA, CotCB, CotE), they were defined as potential antigenic substances for vaccine creation [65, 70, 99–101]. In this group of proteins, special attention was paid to the collagen-like exosporium proteins BclA which form hairy filament structures [69, 70, 73]. *C. difficile* genomes encode three collagen-like BclA paralogs (BclA1, BclA2, BclA3) [65] which are localized only in the exosporium of the *C. difficile* spores [70, 73]. Taking into account that BclA2 and BclA3 are widely presented in the majority of the *C. difficile* strains, it may be supposed that vaccines on their basis will be able to provide immune defense against clinically significant isolates.

An important component of clostridium exosporium is CdeC and CdeM proteins rich in cysteine [10, 30, 64]. CdeM is encountered exclusively in *C. difficile* species [10] while CdeC was detected in other representatives of the *Peptostreptococcaceae* family [30]. When these proteins are not available, the bacterial pathogenicity is decreased [10].

The experiments have proved immunogenicity of the enumerated structural exosporium proteins [30, 64, 102, 103]. The organism of mice which were injected with CdeM protein actively produced IgG-antibodies and developed immune response [103]. Post-vaccination defense after contamination with *C. difficile* reached 90%. Hamsters were vaccinated with CdeM protein isolated from the recombinant strain 630 *C. difficile*. Its efficacy was 80% [102–104]. Investigations in this direction are being carried out taking into consideration the uniqueness of the CdeM protein for *C. difficile*. In particular, the efficacy of the constructed vaccine supplemented by adjuvants to accelerate and prolong its action is being studied [102, 105—109].

In addition to CdeM, another *C. difficile* protein, CdeC, is also used for immunization of laboratory animals. Activation of humoral immunity with the production of IgG was achieved after 3-fold vaccination [103]. Consequently, this protein is also immunogenic [3, 64]. Protection against *C. difficile* infection (strains 630, UK1) after vaccination and the survival of the immunized animals reached 100% [9, 69].

### The role of exosporium in the pathologic process.

The main object in the investigation of the role of exosporium in the infectious process is antropozoonotic agent of anthrax, *B. anthracis*, capable of forming spores under aerobic conditions [14, 84, 89–94].

The initial stage of organism contamination occurs, to the large extent, with an active participation of exosporium, i.e. during the interaction of its superficial collagen BclA glycoprotein with the integrin Мас-1 (CR3) [26, 60, 110]. When the agent spores enter the host organism, they are absorbed by macrophages and dendrite cells which initiate clinical manifestations and the form of the disease (pulmonary, cutaneous, or gastrointestinal) [89, 90, 100, 111, 112]. In any disease form, phagocytes migrate with the lymph flow generating the process. Spores germinate inside the phagocytes and dendrite cells, multiply, and produce toxins. In the lymphatic nodes cell lysis and release of the vegetative bacterial forms take place with subsequent invasion to the blood flow, active proliferation, and toxin production mediating clinical manifestations of the infection and resulting in lethal outcome. The next stage of spore formation goes on in soil only, where there is enough oxygen which is the inductor of sporulation [78, 79, 110].

Thus, a key link in the infectious process is binding of the causative agent spore to integrin and their phagocytosis. Oliva et al. [110] and Bozue et al. [78] have demonstrated that *B. anthracis* spores deprived of the BclA protein do not bind to the integrin. Rhamnose contained in the BclA was shown to bind to СD14 and to act as a coreceptor during Мас-1 integrin interaction promoting phagocytosis. The data presented indicate to the leading role of the exosporium BclA glycoprotein in the pathogenesis of anthrax.

Despite a significant progress in the understanding of the structure and functions of exospores achieved in recent years, our notion of the composition, mechanisms of their generation and the role of this outer structure of bacterial spores remains insufficient [10, 14, 33, 113]. Obviously, investigations began in the XIX century by R. Koch should be continued and much is to be discovered in relation to the biology of exospores.

Application of the state-of-the-art analytical tools used for studying the microbiology of single cells for the exploration of these structures (atomic-force microscopy, Raman spectroscopy, genetics) may become a key to a more comprehensive understanding of the morphological, nanomechanical, and biochemical characteristic of exosporium [12, 113, 114].

## Modern technologies for identification and investigations of spores

### Atomic-force microscopy: from visualization to atomic manipulation.

Scanning probe microscopy is one of the most dynamically developing analytical technologies actively used in current scientific research. One of the most known instruments is an atomic-force microscope invented in 1986 as a scanning tunneling profilometer [28]. Since the 90s of the last century, the atomic-force microscopy (AFM) has been used most actively in biomedical investigations owing to a simple procedure of sample preparation and the capability of submicron visualization of the objects [28, 115—118]. Within a short period of time, AFM as a method of 3D imaging and examining local micromechanical properties has won the leading positions in various fields of science including microbiological investigations [28, 116, 119]. This analytical instrument has supplemented and broadened the potential of the visualization method which is basic for microbiologists, i.e. light microscopy, going beyond its technical resolution, and electron microscopy with its main drawback consisting in the complexity of sample preparation and the necessity to investigate under conditions of a high vacuum [116, 120, 121].

Atomic-force microscopy is attractive for microbiology due to some specific technical features of this diagnostic method based not on the lens properties but rather on the application of a special probe (sensor) and flexible resilient cantilever mounted in the holder.

This imaging instrument combines microscopy in its usual form with nanomolecular detection of mechanical, immunochemical, adhesive, and electrostatic properties of the object (bacteria). These capabilities made AFM an extremely useful and indispensable tool in molecular microbiology, and the prokaryote cells owing to their size and properties became an advantageous object for the study [116, 122, 123]. As a result of scanning, AFM provides a digital three-dimensional topographic image of the microorganism surface in the nanometer lateral and special resolution in any medium and at different temperatures [121, 123].

During a short period of time, AFM turned from the method of topographic imaging into the instrument of separate atom manipulation and investigation of molecular interaction. The capabilities of AFM in the study of bacterial spores will be considered below.

Periodic atomic-force observations of bacterial endospores allow tracking the dynamic processes connected with sporulation or germination into the vegetative cells. It is essential for the microbiologic investigations that AFM can be performed in several modes. In the topographic mode (“constant force”) the displacement of the cantilever is maintained constant using a feedback loop. The force of stylus interaction with the sample surface depends considerably on the distance between them and is determined by adhesion and also by the Van der Waals and capillary interactions. Z-position of the cantilever reflects the sample topography (the height) like a geographic map where the color of the image corresponds to the relief height [116, 118, 123].

Being the method of the force probing, AFM allows one not only to obtain the image of the surface topography and multilayer spore architecture but to determine their nanomechanical characteristics such as resilience, viscosity, and adhesion [12, 121—123]. It is possible to acquire information on the sample surface topography using Young modulus (modulus of elasticity) and adhesion as a high-resolution image [115, 118, 124]. For example, in the studies performed by Giorno et al. [24, 60] and Brunsing et al. [27] with the help of AFM in the aqueous solutions, it has been found that the surface of the *Bacillus spp.* spores has a number of round ribbed nanometer projections on the outer coat oriented along the longitudinal axis, while Zolock et al. [125] showed that based on these morphological features four closely related species of *Bacillus* spores can be differentiated.

Visualization of the spores in a liquid opens wide perspectives for the monitoring of dynamic processes such as sporulation and germination of the dormant cellular forms. Recent interesting examples are the studies of the germination dynamics of *Bacillus spp.* spores into the vegetative cells. After 30 min incubation at room temperature, the spore size increase (swelling) was observed and 5–6 h later the spore outgrew into the vegetative cells which immediately formed a biofilm [121, 123].

Modern capabilities of using AFM for studying the nanomechanical properties of the *B. anthracis* spore inner structures have been demonstrated by Li et al. [12] and Morisaku et al. [123]. On the basis of the thermoprobe AFM (scanning thermal microscopy, SThM) they developed a technique of nanosurgical cutting of the spore using a hard diamond tip while a soft probe was applied for visualization and characteristic of its inner structure in the nanometer scale. It has been found that values of elasticity and adhesion at high temperatures vary in different regions of the spore section [123], there also was detected a previously unknown peptidoglycan ultrastructure of the *B. anthracis* spore cortex consisting of the rod-shaped nanometer structures oriented in the transverse direction relative to the longitudinal spore axis [12, 123].

For almost a 30-year history of employment in the molecular microbiology, force microscopy became a powerful research tool in the exploration of spores effectively supplementing light microscopy, genetics, and biochemical methods traditionally used to analyze the cellular wall structure of dormant bacterial forms. However, the potential application of this technique in the study of dormant bacterial cellular forms is still underestimated and is limited only by some of its functional capabilities [114, 126]. Further application of AFM is connected with the employment of dynamic (multifrequency, multiharmonic, bimodal) techniques which allow one to obtain faster quantitative nanomechanical characteristics of the complex multilayer spore structures with a higher resolution [123, 127].

Interesting and promising is the development and application of AFM biosensors for the quantitative visualization of separate molecules of endospores and exosporium structural proteins using G-quadruplex DNA technologies or functionalized probes with immobilized chemical groups of single molecules especially in combination with other analytical tools such as Raman scattering spectroscopy [126–128].

### Raman spectroscopy: new capabilities of the old technique.

The development of highly sensitive and fast methods of detecting bacterial spores is a challenging task for medical diagnosis, epidemiology, and for solving the tasks of biosafety.

A public outcry caused by the bioterrorist attack in 2001 in which the spores of *B. anthracis* were used has sharpened the problem of their long detection and identification. It was necessary to grow the material in the nutrient media till the vegetative cellular forms and biomass accumulation, and finally to conduct molecular genetic investigations in order to reveal *16S* рRNA gene specific for this type of pathogen. Besides, similar investigations lasting several hours have to be conducted with other suspicious powders to evaluate the scale of the assault [79, 129, 130]. As a result, it was recognized that new informative and more rapid detection methods are required. Early information on pathogen identification would have accelerated the beginning of specific preventive measures, etiopathogenetic treatment, and would have minimized tragic consequences of the terrorist attack caused by the number of infected and died people [27, 131, 132].

The problem of rapid identification is complicated by some aspects connected with the minimal amount of the examined material (inhaling only 10^4^ of *B. anthracis* endospores (100 ng of powder) is a fatal dose for 50% of the infected people) and with the necessity to differentiate the pathogenic biosubstrate from the similar but harmless substance to eliminate the panic fear accompanying the epidemic outbreaks of dangerous infections [99, 129, 133]. Therefore, a new indication method must be not only fast but highly specific, reliably differentiating dangerous bacterial endospores from other biological objects anywhere in the environment.

Raman scattering spectroscopy (RS) meets these criteria being known over 80 years and widely used for a long time as an analytical and applied tool in chemical and technical branches [52, 86, 117]. Low intensity of the signal obtained from the live object has long been an obstacle for the application of RS in biomedical investigations. However, after the improvement of this technology in the last 10–20 years (primarily the integration of RS with confocal microscope and various means of the scattering signal amplification) it is widely used for biomedical investigations requiring no time for sample preparation, special marking, or staining [117]. This powerful analytical tool has been actively employed in recent years for fast, inexpensive, and effective solution of the tasks in molecular microbiology [52].

**Figure 3:**
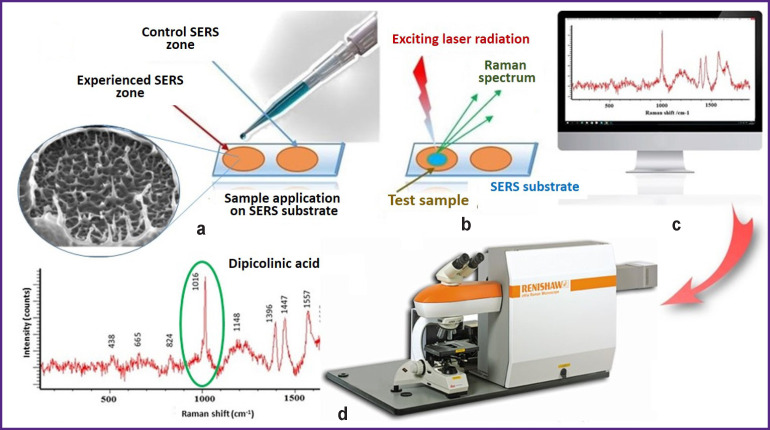
Surface-enhanced Raman spectroscopy (SERS) is the most promising analytical tool for spore indication: (a) application of the sample on the silver or golden SERS-active substrate treated by laser; (b) treatment of the sample by excitation laser beam of a definite wavelength; (c) scattering of Raman rays; (d) recording of Raman spectrum using Raman confocal microscope (photo and drawing courtesy of the authors)

Raman spectroscopy is attractive owing to its noninvasiveness (the capacity to examine bacteria not destroying them), minimal sample preparation, absence of marks and probes, and the rate of obtaining the result. The examined sample is placed in focus of the excitation laser and measured. Molecular information acquired by RS makes it possible to identify unambiguously the microorganism according to the specific spectrum of the chemical and biochemical substances which it is composed of [129, 131, 132]. Besides, the possibility to obtain data on the molecular structure of the bacterial cells which are at different stages of their life cycle as well as to study the dynamics of some cellular processes made this technique a powerful analytical tool for spore exploration (Figure 3).

Significant differences in the spectra of the Raman scattering for the vegetative and spore cellular forms of bacteria were detected relatively long ago investigating *Bacillus spp*. These differences are related to the predominance of calcium dipicolinate in the resonance spectrum of the bacterial endospores whose major peaks were found at the oscillation frequencies of 1015–1017 cm^–1^ and 244 nm excitation wavelength [22, 43, 44, 47, 133]. As pointed above, the content of this marker amounted on average to 10% of a dry spore mass and vary dependent on the species, spore-forming bacterial strain, and sporulation conditions [37, 40, 43, 47]. The obtained Raman spectra of *B. cereus*, *B*. *anthracis*, and *B. subtilis* spores appeared to be very similar and calcium dipicolinate was proposed as a sensitive biomarker for a rapid detection of *Bacillus spp*. spores [134–136].

In recent years, investigations of bacterial spores are carried out using improved RS modifications amplifying the signal (employment of the resonance excitation frequency, laser optic forceps, silver and gold nanoparticles, or coherent anti-Stokes Raman scattering) [135]. Thus, Wang et al. [1, 137] studied the kinetics and levels of accumulation of berberine alkaloid in the *Bacillus spp*. and *Clostridium spp*. spores and its effect on their germination. The aim of the study was to find the means of minimizing potential danger of the germinated spores and the methods of their elimination after or during their germination. In this work, RS laser forceps in combination with fluorescence and differential interference microscopy were utilized to analyze localization, level, and kinetics of berberine absorption in a separate dormant and germinating spore.

The combination of AFM and RS techniques is thought to be very perspective for the investigations of bacterial spores. These integrated analytical systems have been used in molecular microbiology in recent years [48, 117]. Information about the structural topography and nanomechanical properties is supplemented with the data about a submicron molecular profile which gives a fairly complete picture and better understanding of the dormant systems [117]. For example, Boitor et al. [138] have evaluated quantitatively RNA, DNA, and proteins of the bacterial cellular forms by recording the Raman spectra and calibrating models for each structural component and correction of focus effects using AFM topographic images.

One of the latest modifications of this method, the combination of RS with confocal microscopy, contributed to the appearance of a powerful instrument for the study of bacterial spores, micro Raman spectroscopy (microRS). This perspective technique renders a unique possibility for the investigation of separate cells and cellular elements. Recent investigations of the bacterial spores of the species of *Bacillus* genus using microRS [137–139] made it possible to obtain interesting findings on their phenotypic heterogeneity related to the environmental parameters and also to test individually methods of inactivation of these cellular forms. Sporicidal capability of some physical and chemical methods was evaluated at the level of separate spores and, ultimately, treatment with 20% formaldehyde appeared to be most effective [138]. Besides, the cold atmospheric plasma has been shown to inactivate the spores highly effectively and prospectively in the medical and food industries [137, 139].

In recent years, spectroscopic methods for the bacterial spore investigations attracted attention as the main element for the combination with other analytical instruments [52, 117, 139]. This interdisciplinary approach reflects a new trend and is an indispensable research technology of multiparametric exploration of heterogeneity of single cells in microbiology (a paradigm of unicellular microbiology) including bacterial spores. And in this regard, AFM and RS serve as examples of novel analytical tools and techniques which render a unique possibility to observe discrete microbiological phenomena unavailable which traditional approaches are not able to realize.

## Plasmochemical technologies and bacterial spores

Bacterial spores have been known since the emergence of microbiology as a science. For a long time, they have been considered from the point of view of their epidemiologic and pathogenetic importance for the occurrence of infections. As mentioned above, extreme stability in the environment made bacterial spores a serious problem of public health. The existing sterilization methods (dry and wet steam, chloride-containing substances, γ-irradiation, and others) are not sufficiently effective especially during decontamination of surgical instruments, medical equipment, or pharmaceuticals. Therefore, the search for the means and methods of bacterial spore elimination was and remains a challenging scientific task which is being solved by involving numerous interdisciplinary investigations.

To realize this goal, the possibility of using nonthermal ambient-temperature (cold) plasma is actively studied since it may be employed for the treatment of thermosensitive objects and is considered as a potential alternative to the traditional sterilization methods [137, 139, 140]. Such plasma is generated by the exposure of gas (some inert gas, for example) to electric or electromagnetic fields. The field energy causes acceleration of free electrons and ionizes the gas atoms and molecules. Excited atoms and molecules returning to the more stable state release excess energy in the form of electromagnetic or ultraviolet radiation and as active forms of oxygen and nitrogen with a wide and powerful antimicrobial spectrum of action [141–143]. The most studied applications of this technology in biology and medicine are food sterilization, decontamination of medical equipment, surgical implants, and treatment of wound surfaces [142, 144—146].

Rapid (seconds/minutes) and effective effect of the cold plasma on dormant and vegetative cellular forms of pathogenic gram-negative and gram-positive bacteria including *Bacillus spp*. and *Clostridium spp*. spores attracted great interest of microbiologists [145, 147, 148]. It was focused primarily on the study and characteristic of plasma antimicrobial effectivity and mechanisms of microbial inactivation and sporicidal action which are presently being actively studied [141, 149, 150].

## Conclusion

Bacterial spores are a unique and highly inert biological system with mechanisms of resistance to the extreme conditions which are not completely studied. Understanding the mechanisms of stability of these dormant forms is of great biomedical importance and can potentially lead to the development of novel methods of prophylaxis and sterilization which will be directed to the vulnerable structures of endo- and exospores.

A long-term history of studying endospores allowed microbiologists to analyze in detail and describe the process of sporulation and germination in separate model organisms, to create a firm basis for understanding the cellular processes leading to the generation of these dormant forms, to become partially aware of the diverse mechanisms of their tolerance. The result of the investigations became a conceptual persuasion that high stability of the bacterial spores may be important and find application in various fields of biotechnology.

Presently, the exploration of the spores goes on at a new nanostructural level. For instance, spores are of scientific interest as a biological model for studying the mechanisms of generating complicated supramolecular structures and become a target for modern biotechnologies following the principle “to learn from nature” [150–152]. Very perspective are the developments of the models of endospore natural architectonics for creation of self-organizing supramolecular structures used as nanodimensional substrates for packing and target delivery of enzymes, nucleic acids, antigens, and drugs to the definite tissues, cells and even intracellular organelles [153–155]. These surfaces may be used as biocoatings or molecular switchers activated by chemical substances, electrons, or light [156, 157].

A direct utilization of endospore is also considered though there is a significant risk of germination and vegetative growth of bacteria before they reach the target tissue. Nevertheless, Roberts et al. [154] used endospores of *C. histolyticum* and *C. novyi* in the experiment and found that these species caused lysis of the tumor cells or tumor regression during germination and vegetative growth.

Another direction connected with the application of spores in the treatment of malignant tumors is a selective expression of specific enzymes in the predominant germination of dormant in hypoxic tumors [155].

From the beginning of the XXI century, researches have been carried out on the usage of recombinant bacterial spores for creation of thermostable vaccines [149, 151, 158–160]. In 2007, Uyen et al. [153] conducted a successful experiment on protection of mice against tetanus by oral and nasal introduction of the vaccines constructed on the bases of antigen *C. tetani* TTFC on the surface of *В. subtilis* spores. The conducted pilot studies proved the prospectivity of these biotechnologies [156, 157, 161].

It has been supposed in recent publications [10, 36, 152, 162] that spores and spore-forming bacteria may play an important role in the development and spread of resistance to antibiotics due to their biological properties, ability to dissemination and thereby to the propagation of resistance genes to antimicrobial substances in the state of metabolic rest [10, 36, 152].

In the nearest future, the unique properties of bacterial spores are believed to find their application in other ecosystemic technologies, for example, in paleoecologic probing, as biofungicides and bioinseticides in agriculture, as well as during bioremediation and biomineralization (biologically induced and controlled processes of mineralization that go on in nature).
